# Seroepidemiology of Hepatitis E Virus Infection in Mennonites in Mexico

**DOI:** 10.14740/jocmr1993w

**Published:** 2014-11-19

**Authors:** Cosme Alvarado-Esquivel, Luis Francisco Sanchez-Anguiano, Jesus Hernandez-Tinoco

**Affiliations:** a Biomedical Research Laboratory, Faculty of Medicine and Nutrition, Juarez University of Durango State, Durango, Mexico; b Institute for Scientific Research “Dr. Roberto Rivera Damm”, Juarez University of Durango State, Durango, Mexico

**Keywords:** Hepatitis E virus, Ethnic groups, Seroepidemiologic studies, Rural population, Case-control study, Mexico

## Abstract

**Background:**

The seroepidemiology of hepatitis E virus (HEV) infection in Mennonites has not been studied. We aimed to determine the seroprevalence of anti-HEV IgG antibodies in Mennonites in Durango, Mexico, and to compare it with the seroprevalence in general population in rural Durango. The socio-demographic, clinical and behavioral characteristics of Mennonites associated with HEV seropositivity were also investigated.

**Methods:**

We performed a case-control study to determine the frequency of anti-HEV IgG antibodies in 150 Mennonites (mean age 38.40 ± 15.53 years old) and 150 age- and gender-matched non-Mennonites controls using an enzyme-linked immunoassay. We used a standardized questionnaire to obtain the socio-demographic, clinical and behavioral characteristics of the Mennonites.

**Results:**

Anti-HEV IgG antibodies were detected in 10 (6.7%) of 150 Mennonites and in 61 (40.7%) of 150 controls. Seroprevalence of anti-HEV IgG antibodies was significantly lower in Mennonites than in controls (odds ratio (OR) = 0.009; 95% confidence interval (CI): 0.0006 - 0.15; P < 0.000001). Logistic regression of socio-demographic and behavioral characteristics of Mennonites showed that HEV seropositivity was only associated with increasing age (OR = 1.05; 95% CI: 1.00 - 1.09; P = 0.03). While sex, birth place, residence, educational level, socio-economic status, occupation, animal contacts, foreign travel, frequency of eating away from home, consumption of raw or undercooked meat, type of meat consumed, consumption of unpasteurized milk or untreated water, and consumption of unwashed raw vegetables or fruits were not associated with HEV seropositivity. None of the Mennonites suffered from clinical hepatitis.

**Conclusions:**

Results demonstrate: 1) serological evidence of HEV exposure in Mennonites; however, Mennonites have a lower seroprevalence of HEV antibodies than controls from the rural general population; 2) seroprevalence in Mennonites increased with age. Further studies with a larger sample size to determine more contributing factors for HEV infection in Mennonites are needed.

## Introduction

Hepatitis E virus (HEV) causes infections worldwide regardless of the development of the countries [1, 2]. HEV is considered the most frequent pathogen responsible for acute viral hepatitis in the world [3]. HEV can be transmitted by a number of routes including fecal-oral transmission [4, 5], blood transfusion [6, 7], and consumption of meat from HEV-infected animals, i.e., pork, wild boar and deer [8-10]. Infections with HEV have caused outbreaks especially in developing countries by drinking water contaminated with feces [10-12]. Infections with HEV usually lead to an acute self-limited disease [13], but fulminant acute hepatitis may occur [14]. HEV causes a low clinical illness rate in children but a high fatality rate in pregnant women [5]. In immunocompromised individuals, HEV may cause chronic infection with rapidly progressive disease [13, 14]. In addition, HEV infection is responsible for mortality in patients with underlying chronic liver disease [6].

The seroepidemiology of HEV infection in ethnic groups in the world has been poorly studied. There is a lack of information about the seroepidemiology of HEV infection in Mennonites (an ethnic group of Mexican citizens of German descent or origin). Mennonites in Durango, Mexico live in rural areas and work usually in agriculture and livestock raising. We sought to determine the seroprevalence of anti-HEV IgG antibodies in Mennonites in Durango, Mexico, and to compare it with the seroprevalence in general population in rural Durango. The socio-demographic, clinical and behavioral characteristics of Mennonites associated with HEV seropositivity were also investigated.

## Methods

### Study design and study population

Through a case-control study design using archival serum samples, we compared the seroprevalence of HEV infection in 150 Mennonites and 150 age- and gender-matched people of the general population in rural Durango, Mexico. Archival serum samples were originally used to determine the seroepidemiology of *Toxoplasma gondii* infection in Mennonites [15] and general population [16] in rural Durango, Mexico. The latter population was also recently studied to determine the epidemiology of HEV infection in rural Durango, Mexico [17]. Inclusion criteria for the Mennonites (cases) were: Mennonites living in Nuevo Ideal, Durango, aged 17 years and older and who accepted to participate in the study. Exclusion criteria for Mennonites were: insufficient amount of serum and incomplete socio-demographic data. In total, 150 Mennonites (90 male and 60 females) were included in the study. They were 17 - 85 (38.40 ± 15.53) years old. Inclusion criteria for the general population of rural Durango (controls) were: inhabitants of rural Durango, aged 17 years and older, and who accepted to participate in the study. Exclusion criteria for controls were the same as in cases. Controls were randomly selected and included 79 males and 71 females aged 18 - 91 (40.83 ± 18.81) years old. Cases and controls had comparable number of males and females (P = 0.20) and age (P = 0.22).

### General characteristics of Mennonites

We used a standardized questionnaire to obtain the socio-demographic, clinical, and behavioral characteristics of the Mennonites. Socio-demographic items included age, sex, birth place, residence, educational level, socio-economic status, and occupation. Clinical data included health status, history of blood transfusions or transplants. Behavioral characteristics included animal contacts, foreign travel, frequency of eating away from home (in restaurants or fast food outlets), consumption of raw or undercooked meat, type of meat consumed (pork, lamb, beef, goat, boar, chicken, turkey, rabbit, venison, squirrel, horse or other), consumption of ham, sausages or salami, and hygiene practices as consumption of unpasteurized milk or untreated water, and consumption of unwashed raw vegetables or fruits.

### Laboratory tests

Serum samples were analyzed for anti-HEV IgG antibodies by a commercially available enzyme immunoassay “HEV-IgG ELISA” kit (Diagnostic Automation Inc., Calabasas, CA, USA). According to the manufacturer, this assay has a sensitivity of 99.8% and a specificity of 99.8%. All immunoassays were performed following the manufacturer’s instructions, and negative and positive controls were tested in each assay.

### Statistical analysis

We used the software Microsoft Excel, Epi Info version 3.5.4 and 7, and SPSS version 15.0 for the statistical analysis. For calculation of the sample size, we used a 95% two-sided confidence level, a power of 80%, a 1:1 ratio of cases and controls, a reference seroprevalence of 6.3% [18] as the expected frequency of exposure in controls, and an odds ratio of 3. The result of the sample size calculation was 145 cases and 145 controls. Age in cases and controls was compared by the paired Student’s *t*-test. We used the McNemar’s paired test for comparison of the HEV seroprevalence in cases and controls. In addition, we used the Pearson’s Chi-squared test and the two-tailed Fisher’s exact test (when values were small) for an initial assessment of the association of HEV seropositivity and Mennonites’ characteristics. Socio-demographic and behavioral characteristics of the Mennonites with a P value < 0.35 obtained in the bivariate analysis were further analyzed by multivariate analysis using backward stepwise logistic regression analysis. Variables with zero values were not included in the multivariate analysis. Odds ratio (OR) and 95% confidence interval (CI) were calculated by the Mantel-Haenszel analysis. Statistical significance was set at a P value less than 0.05.

### Ethical aspects

We used only archival serum samples and data from two previous surveys [15, 16]. Both surveys were approved by the Institutional Ethical Committees of the Mexican Social Security Institute and the General Hospital of the Secretary of Health in Durango City. The purpose and procedures of the surveys were explained to all participants, and a written informed consent was obtained from all of them.

## Results

Anti-HEV IgG antibodies were detected in 10 (6.7%) of 150 Mennonites and in 61 (40.7%) of 150 controls. Seroprevalence of anti-HEV IgG antibodies was significantly lower in Mennonites than in controls (OR = 0.009; 95% CI: 0.0006 - 0.15; P < 0.000001) (Table 1).

**Table 1:** Distribution of HEV Infection Among Case-Control Pairs

Case	Control		Total	OR (95% CI)	P-value
	Exposed (HEV infection)	Unexposed (no HEV infection)			
Exposed (HEV infection)	10	0	10		
Unexposed (no HEV infection)	51	89	140		
Total	61	89	150	0.009 (0.0006 - 0.15)	< 0.000001

0.5 has been added to each cell for calculations.

Of the socio-demographic characteristics of Mennonites (Table 2), three variables showed P values < 0.35 by bivariate analysis: sex (P = 0.09), age (P = 0.12), and occupation (P = 0.09). Other socio-demographic characteristics including birthplace, residence, educational level, and socio-economic status had P values > 0.35 by bivariate analysis.

**Table 2:** Socio-Demographic Characteristics of Mennonites and Seroprevalence of HEV Infection

Characteristic	No. of subjects tested^a^	Prevalence of HEV infection		P value
		No.	%	
Gender				
Male	90	3	3.3	0.09
Female	60	7	11.7	
Age groups (years)				
30 or less	58	1	1.7	0.12
31 - 50	59	5	8.5	
> 50	33	4	12.1	
Birth place				
Durango State	137	9	6.6	0.48
Other Mexican State or abroad	9	1	11.1	
Residence place				
Durango State	144	10	6.9	1
Abroad	3	0	0.0	
Educational level				
No education	14	0	0.0	0.54
1 - 6 years	116	9	7.8	
7 or more years	17	1	5.9	
Occupation				
Laborer^b^	95	4	4.2	0.09
Non-laborer^c^	51	6	11.8	
Socio-economic level				
Low	18	2	11.1	0.36
Medium and high	126	8	6.3	

^a^Sums may not add up to 150 because of missing values. ^b^Employee, factory worker, business, student, professional, other, none. ^c^Housewife, student, none occupation.

With respect to the behavioral characteristics assessed, the variables consumption of pork, beef, pigeon meat, ham, unwashed raw fruits, and untreated water as well as eating away from home and traveled abroad had P values < 0.35 by bivariate analysis (Table 3). Other behavioral characteristics including animal contacts, consumption of raw or undercooked meat, consumption of meat other than pork, beef, or pigeon meat, consumption of sausages or salami, unpasteurized milk or unwashed raw vegetables had P values > 0.35 by bivariate analysis. The variable consumption of unwashed raw fruits had a zero value in the bivariate analysis and was not evaluated in the multivariate analysis. Further analysis using logistic regression of socio-demographic and behavioral characteristics of Mennonites showed that HEV seropositivity was only associated with increasing age (OR = 1.05; 95% CI: 1.00 - 1.09; P = 0.03).

**Table 3:** Bivariate Analysis of a Selection of Behavioral Characteristics of Mennonites and HEV Infection

Characteristic	No. of subjects tested^a^	Prevalence of HEV infection		P value
		No.	%	
Raising animals^b^				
Yes	124	8	6.5	0.64
No	22	2	9.1	
Pork meat consumption				
Yes	132	8	6.1	0.24
No	14	2	14.3	
Beef consumption				
Yes	141	9	6.4	0.3
No	5	1	20	
Pigeon meat consumption				
Yes	38	4	10.5	0.29
No	107	6	5.6	
Duck meat consumption				
Yes	31	3	9.7	0.44
No	114	7	6.1	
Degree of meat cooking				
Undercooked	8	1	12.5	0.44
Well done	136	9	6.6	
Raw cow milk consumption				
Yes	86	7	8.1	0.74
No	58	3	5.2	
Sausages consumption				
Yes	138	9	6.5	0.4
No	7	1	14.3	
Ham consumption				
Yes	132	8	6.1	0.22
No	13	2	15.4	
Salami consumption				
Yes	59	5	8.5	0.52
No	86	5	5.8	
Unwashed raw fruits				
Yes	31	0	0	0.11
No	113	10	8.8	
Untreated water				
Yes	86	8	9.3	0.31
No	58	2	3.4	
Traveled abroad				
Yes	84	8	9.5	0.19
No	62	2	3.2	
National trips				
Yes	107	8	7.5	0.44
No	38	1	2.6	
Frequency of eating out of home				
Up to 10 times a year	74	7	9.5	0.16
More than 10 times a year	70	2	2.9	

^a^Sums may not add up to 150 because of missing values. ^b^Raising of any kind of animals.

Concerning clinical characteristics, seroprevalence of HEV infection was similarly observed in ill (3/31: 9.7%) and healthy (6/113: 5.3%) Mennonites (P = 0.40), and none of the Mennonites was suffering from clinical hepatitis. In addition, none of the Mennonites with history of blood transfusion or transplantation had anti-HEV IgG antibodies.

## Discussion

There is a poor knowledge on the seroepidemiology of HEV infection among ethnic groups in the world. The present study was performed to investigate the presence of HEV infection in a sample of Mennonites in rural Durango, Mexico. We found that HEV infection exits in Mennonites but in a low seroprevalence rate. The seroprevalence of HEV infection in Mennonites (6.7%) was significantly lower than the seroprevalence found in the gender- and age-matched controls (40.7%). The low seroprevalence of HEV infection in Mennonites was unexpected. Mennonites live in rural communities and an increased (36.6%) seroprevalence of HEV infection has been found in general population in rural Durango [17]. It is not clear why Mennonites have a lower seroprevalence of HEV infection than the one in general population in rural Durango. Both populations groups reside in rural communities, have comparable age and number of males and females, and have contact with animals. We are not aware of further studies on the seroepidemiology of HEV infection in Mennonites or any other ethnic group in Mexico. Therefore, the HEV seroprevalence found in Mennonites cannot be compared with other seroprevalence rates in ethnic groups in the country. There have been two previous studies on the seroepidemiology of HEV infection in rural Durango State. One study included pregnant women [19] and the other study included general population [17]. However, seroprevalences found in such studies are not suitable for comparison with the seroprevalence found in Mennonites because of the lack of gender and age controlling. In the national context, there have been two previous seroepidemiological studies about HEV infection. However, seroprevalences found in such studies cannot be compared with that in Mennonites because of differences in serological assays and characteristics of the population groups among the studies. In a first study, Bernal-Reyes and Licona-Solis [18] found an HEV seroprevalence of 6.3% in general population in Hidalgo State which is located in central Mexico. In a national survey, researchers found a 10.5% seroprevalence of HEV infection in subjects aged 1 - 29 years old [20]. In contrast, the mean age of the Mennonites studied was 38.90 years old. In the international context, seroprevalence of HEV infection in Mennonites is as low as the 6.0% national seroprevalence in general population in the USA reported recently [21].

Concerning socio-demographic and behavioral characteristics of Mennonites associated with HEV seropositivity, multivariate analysis showed that HEV exposure was only associated with age. Older Mennonites have a higher seroprevalence than younger Mennonites. This finding is consistent with results of other studies where seroprevalence of HEV infection increases with age [4, 21]. No further characteristics of Mennonites were found associated with HEV infection in the present study. The low frequency of HEV infection in Mennonites did not allow us to obtain enough power to reach statistical significance in the analysis of likely contributing factors for HEV infection. Consumption of untreated water and availability of water at home have been associated with HEV exposure in rural Durango [17]. Mennonites with consumption of untreated water had a higher (but not statistically significant) seroprevalence (9.3%) than those without such practice (3.4%). Intriguingly, Mennonites do not obtain water from any public water supplying system but obtain water from their own water wells at home. This is a clear difference among Mennonites and controls. People in rural communities in Durango usually obtain water from pipes of public water supplying systems, and contamination of such system might lead to a wide dissemination of HEV to many houses. The lack of such contributing factor for HEV in Mennonites might explain the low seroprevalence of HEV infection in Mennonites. This fact suggests a reduction in the transmission of HEV infection by using water from individual water wells in each house.

### Conclusions

We conclude: 1) results of the present study demonstrate serological evidence of HEV exposure in Mennonites; however, Mennonites have a lower seroprevalence of HEV antibodies than controls from the rural general population; 2) seroprevalence in Mennonites increased with age. Further studies with a larger sample size to determine more contributing factors for HEV infection in Mennonites are needed.
